# Practice Makes Perfect: Learning Mind Control of Prosthetics

**DOI:** 10.1371/journal.pbio.1000152

**Published:** 2009-07-21

**Authors:** Caitlin Sedwick

**Affiliations:** Freelance Science Writer, San Diego, California, United States of America

**Figure pbio-1000152-g001:**
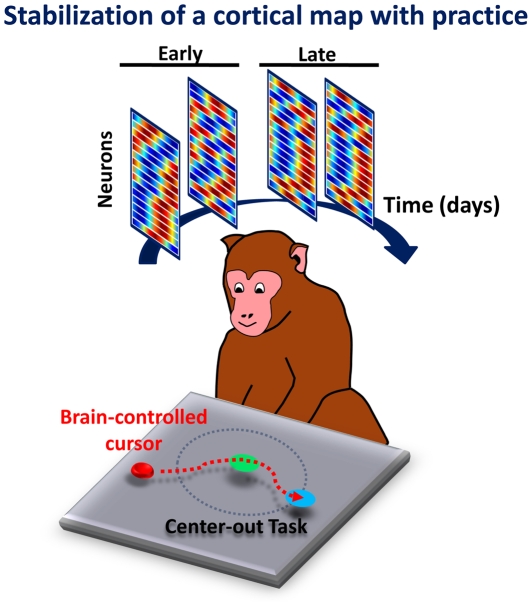
The primate brain is able to form a map for neuroprosthetic control that meets all the essential properties of memory—that is, stable, readily recalled, and resistant to interference.

[Fig pbio-1000152-g001]More than 5 million people in the United States are afflicted with some form of paralysis, leaving them with diminished physical capacity. Fortunately, the past few years have seen advancements in both stem-cell technologies, which promise to repair such injuries, and in the development of sophisticated prosthetic devices that offer patients a way to interact with their environment. For example, recent studies have demonstrated that monkeys and humans can control computer cursors or robotic limbs through electrode arrays implanted in their brains. However, the neuronal changes associated with long-term prosthetic skill acquisition remain unclear. A better understanding of this process is crucial for the reliable control of prosthetic devices in a manner that mimics our effortless control of limb movements. In this issue of *PLoS Biology*, Karunesh Ganguly and Jose Carmena describe experiments that specifically track the neural changes associated with the process of learning and reliably controlling such devices through an implanted electrode array. This work demonstrates that the brain can create a stable mental representation of the prosthetic device that eventually allows for precise control.

To explore how the brain learns to control these prosthetic devices, the authors first needed to create a model of how the brain controls a normal limb. To do this, they trained two monkeys to conduct a simple task—reaching from the center of a circle to a specified target (indicated by a colored light) along the circle's edge. Meanwhile, an array of electrodes implanted in the motor cortex of the animals' brains recorded the electrical activity of nearby neurons as the animals completed the task. This information was correlated with the spatial location of the monkey's hand to derive a mathematical model, or “decoder,” for the relationship between the activity of individual motor cortex neurons and the direction of arm movement. Then, the monkeys were trained to operate a virtual version of the reaching task on a computer screen without moving their arms.

Like a child learning to ride a bike, the monkeys initially found the virtual version of the task difficult to complete; their control of cursor movement was erratic, and it took them a long time to complete the task. But with several days of practice, precise control of the cursor was learned.

What kinds of neuronal changes occurred during learning of the virtual task? Because the authors were recording the activity of each of the single neurons controlling the virtual device, they were able to monitor changes in neuronal activity over a period of days. The researchers focused first on the “preferred direction” of the recorded neurons: the amount of activity exhibited by individual neurons during motion of a limb in a specific direction. For example, the activity in a particular neuron might be most intense when the hand is moving to the right, but practically undetectable when the hand is moving to the left, while a different neuron may act in an opposite manner.

Ganguly and Carmena recorded the preferred directions of the motor cortex neurons during the manual version of the reaching task on the first day of the experiment, and on following days during the virtual task. They found that the preferred directions of individual neurons actually shifted during the early days of the experiment as the monkeys became accustomed to the virtual device. But, as the animals became more proficient at their task, the preferred directions of individual neurons stabilized into a characteristic pattern, representing a mental map of the device.

The researchers used the same decoder algorithm on each day to translate neuronal activity in the animals' brains into cursor movement. Small changes in the decoder actually hindered the stabilization process, indicating that learning occurs best when the decoder is stable.

The investigators also wondered if more than one mental map could be formed and stored in the brain. To find out, they introduced a new decoder for a monkey to use in place of the old, well-learned decoder. Given several days to practice, the animal gained proficiency with each decoder. Proficiency with the new decoder was associated with a totally distinct pattern of preferred directions than for the original decoder. What's more, the monkey could easily switch back and forth between the different decoders when given a visual cue that the decoder had changed, indicating that the mental maps for the different decoders don't interfere with each other.

Collectively, the authors' findings argue that the primate brain can create a stable mental map for a virtual device and recall it on demand, a process not unlike the natural recall of motor memories. The primates can learn multiple decoders, and, from the brain's perspective, learning to control the device through new decoders is like trying to learn to ride a bike when you're given a radically different bike for every attempt. Learning can happen under these circumstances, but not quickly; one needs practice to perfect the skill.

But once a device is mastered the motor memory persists, perhaps for life.

**Ganguly K, Carmena JM (2009) Emergence of a Stable Cortical Map for Neuroprosthetic Control. doi:10.1371/journal.pbio.1000153**

